# Diversity in boron toxicity tolerance of Australian barley (*Hordeum vulgare* L.) genotypes

**DOI:** 10.1186/s12870-015-0607-1

**Published:** 2015-09-26

**Authors:** Julie E. Hayes, Margaret Pallotta, Melissa Garcia, Mehmet Tufan Öz, Jay Rongala, Tim Sutton

**Affiliations:** Australian Centre for Plant Functional Genomics and The University of Adelaide, Adelaide, South Australia Australia; Present address: University of Florida Agronomy Department, Gainesville, Florida USA; Present address: South Australian Research and Development Institute, Adelaide, South Australia Australia

## Abstract

**Background:**

Boron (B) is an important micronutrient for plant growth, but is toxic when levels are too high. This commonly occurs in environments with alkaline soils and relatively low rainfall, including many of the cereal growing regions of southern Australia. Four major genetic loci controlling tolerance to high soil B have been identified in the landrace barley, Sahara 3771. Genes underlying two of the loci encode the B transporters *HvBot1* and *HvNIP2;1*.

**Results:**

We investigated sequence and expression level diversity in *HvBot1* and *HvNIP2;1* across barley germplasm, and identified five novel coding sequence alleles for *HvBot1*. Lines were identified containing either single or multiple copies of the Sahara *HvBot1* allele. We established that only the tandemly duplicated Sahara allele conferred B tolerance, and this duplicated allele was found only in a set of nine lines accessioned in Australian collections as Sahara 3763–3771. *HvNIP2;1* coding sequences were highly conserved across barley germplasm. We identified the likely causative SNP in the 5’UTR of Sahara *HvNIP2;1*, and propose that the creation of a small upstream open reading frame interferes with HvNIP2;1 translation in Sahara 3771. Similar to *HvBot1*, the tolerant *HvNIP2;1* allele was unique to the Sahara barley accessions. We identified a new source of the 2H B tolerance allele controlling leaf symptom development, in the landrace Ethiopia 756.

**Conclusions:**

Ethiopia 756, as well as the cultivar Sloop Vic which carries both the 2H and *HvBot1* B tolerance alleles derived from Sahara 3771, may be valuable as alternative parents in breeding programs targeted to high soil B environments. There is significant diversity in B toxicity tolerance among contemporary Australian barley varieties but this is not related to variation at any of the four known B tolerance loci, indicating that novel, as yet undiscovered, sources of tolerance exist.

**Electronic supplementary material:**

The online version of this article (doi:10.1186/s12870-015-0607-1) contains supplementary material, which is available to authorized users.

## Background

High soil boron (B) can affect yields of barley (*Hordeum vulgare* L.) across southern Australia by up to 17 % [1], depending on a multitude of site, seasonal and genetic factors [2–5]. Along with disease ratings, standard information for new barley varieties released in South Australia, Victoria and Western Australia often includes a boron tolerance rating [6, 7], allowing farmers to select varieties tolerant to high soil B. Genetic variability for high B tolerance has long been known [8, 9]. The most tolerant barley identified amongst breeding material in Australia is the unadapted six-row North African landrace, Sahara 3771. This genotype was accessioned in Australian collections in the early 1900s [10], one of a set of nine barley lines listed as Sahara 3763 – Sahara 3771. It has been considered an important source of B tolerance for barley breeding programs over many years. Four major QTL for B tolerance were identified in Sahara 3771, in a genetic study using a doubled haploid (DH) population derived from a cross between the South Australian malting variety, Clipper, and Sahara 3771 [11]. Subsequent research to fine-map two of the regions revealed the identity of the tolerance genes *HvBot1* (chromosome 4H) [12] and *HvNIP2;1* (chromosome 6H) [13]. They encode two types of transporter that function to minimise the amount of B in barley roots. These genes have been partially characterised, but the prevalence of the tolerant alleles across Australian germplasm was not known. It was also not known if there is significant diversity in *HvBot1* and *HvNIP2;1* contributing to B tolerance, such as has been found in wheat for *TaBot-B5* [14]. Therefore, the aims of this study were to: 1) determine the prevalence of known B tolerance alleles in Australian barley germplasm; 2) develop an improved set of markers for tracking the introgression of B tolerance from Sahara 3771; and 3) identify alternative sources of B tolerance in barley. This was a broad study and, although the set of germplasm assessed was not exhaustive, our data suggest that the tolerance alleles found in Sahara 3771 are rare. The significance of a QTL on chromosome 2H controlling leaf symptom expression is highlighted as a target for future breeding and selection for B tolerance in barley.

## Results

Tolerance to high soil B in the barley landrace Sahara 3771 has been attributed to four major QTL, on chromosomes 2H, 3H, 4H and 6H. We screened a set of 65 diverse barley genotypes (Additional file 1: Table S1) for variation at these loci using genomic Southern analysis, which also enabled us to assess gene copy number variation. Coding sequence for the genes encoding B transport proteins HvBot1 and HvNIP2;1, and which lie beneath the 4H and 6H tolerance loci, respectively, was also amplified and sequenced. In sourcing diverse germplasm to screen, we obtained seed for nine barleys accessioned in the Australian Grains Genebank as Sahara 3763 to Sahara 3771. Our analyses suggest that the Sahara accessions possess a unique set of B tolerance alleles.

### Genetic variation at the 4H locus (*HvBot1*)

Southern analysis of the 65 barley genotypes revealed that the tandem *HvBot1* gene duplication found in Sahara 3771 is rare, although other genotypes (eg. California Mariout and derivative cultivars CM67 and CM72, and the Japanese cultivars Haruna Nijo and Amagi Nijo) showed a Sahara-like restriction pattern without gene duplication (panel A, Additional file 2: Figure S1). All nine Sahara genotypes from the Australian Grains Genebank possessed the *HvBot1* gene duplication, and displayed similarly high B-tolerant phenotypes in hydroponic experiments (Additional file 2: Figure S1).

**Fig. 1:**
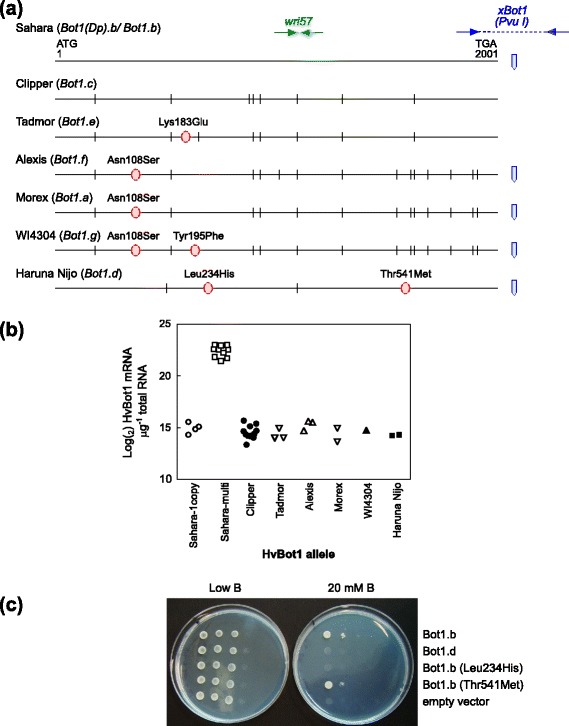
Genetic variation in barley at the 4H B tolerance locus (*HvBot1*). **a** *HvBot1* coding sequence alleles detected in a diverse set of barley genotypes. All SNPs relative to the Sahara allele are shown as lines, with those resulting in an amino acid change shown as circles. The positions of the *xBot1* CAPS marker (*blue*) and the Sahara-specific *wri57* KASP^TM^ assay (*green*) are also indicated. Allele nomenclature (*Bot1.a* to *Bot1.g*) is given according to published guidelines for barley [35]. We designated the allele from the sequencing reference genome of cv. Morex as *Bot1.a*. **b** Expression levels of *HvBot1* determined by q-RT-PCR, in roots of seedlings of barley genotypes carrying different *HvBot1* alleles. Refer to Additional file 8: Table S5 for a list of barley genotypes included in each *HvBot1* allele class. Transcript levels were normalised using the RNA housekeeping genes for tubulin, GAPDH and HSP70. **c** Growth of yeast expressing the Sahara *HvBot1* allele (*Bot1.b*), the non-functional Haruna Nijo *HvBot1* allele containing two residue substitutions relative to Sahara (*Bot1.g*; Leu234His/Thr541Met), and *Bot1.b* with single residue mutations (Leu234His or Thr541Met) relative to an empty vector control, on a low B medium (*left*) or at high B (*right*). Clones are positioned vertically and spots across the plates represent 10 L of 10^−0^, 10^−1^, 10^−2^ and 10^−3^ dilutions from a starting culture containing approximately 3 × 10^7^ cells L^−1^. Photographs were taken after 2 d (*low B*) or 4 d (*20 mM B*) incubation at 30 °C, and are representative of two experiments using independent clones

Sequencing of the coding regions of *HvBot1* revealed seven coding sequence variants (Fig. 1a), including the Clipper and Sahara (single- and multi-copy) *HvBot1* alleles. Eight synonymous SNPs were found between the Clipper and Sahara alleles, with no resulting amino acid changes ([GenBank:EF660437], Clipper *HvBot1* complete CDS, has been updated to reflect this finding). Amino acid substitutions were found in each of the other five variants, at either one (Tadmor, Alexis and Morex) or two (Haruna Nijo and WI4304) of five different locations. The Alexis, Morex and WI4304 variants shared a common substitution (Asn^108^Ser). We have named the allelic variants *Bot1.a* through to *Bot1.g*, as illustrated in Fig. 1a. Coding sequences for the novel alleles have been lodged with GenBank [GenBank:KR605456, GenBank:KR605457, GenBank:KR605458, GenBank:KR605459, GenBank:KR605460].

We compared *HvBot1* expression levels in roots of seedlings of barley genotypes carrying the different alleles (Fig. 1b). *HvBot1* expression was similar for all variants except for genotypes carrying the duplicated Sahara allele (*Bot1(Dp).b*), which in this experiment showed more than 200-fold higher levels of expression (Fig. 1b). Multiple sequencing efforts failed to uncover any sequence differences, in either the *HvBot1* gene or its promoter, between copies in lines carrying the *HvBot1* duplication, or between the duplicated allele and lines containing a single copy of the Sahara *HvBot1* allele.

**Fig. 2:**
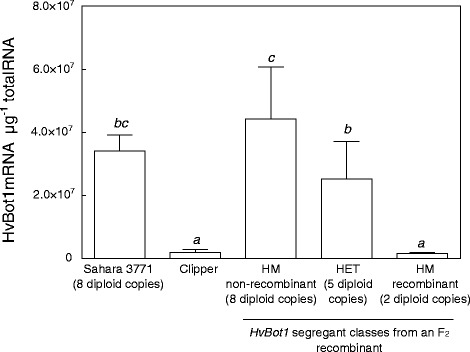
*HvBot1* duplication is correlated with higher levels of *HvBot1* expression. *HvBot1* transcript levels (*N* = 6, ± sd) in barley roots of the parental genotypes Sahara 3771 and Clipper, and in *HvBot1* segregant classes from an F_2_ recombinant derived from a cross between two doubled haploid lines. Italicised letters above the bars denote significant differences between genotypes (Tukey’s multiple comparisons test; *P* = 0.05). The F_2_ was identified as recombinant within the *HvBot1* gene cluster, resulting in F_3_ progeny with different numbers of copies of the Sahara *HvBot1* allele. Southern analysis indicated that the homozygous recombinant progeny contained only a single copy of the Sahara *HvBot1* allele. Seedlings were grown for 14 days in nutrient solution with an additional 2 mM B prior to sampling. F_3_ individuals were genotyped using the closely linked *xBM178* CAPS marker (S3 Table), to identify segregant classes

We identified an individual F_2_ seedling from a cross between two Clipper X Sahara 3771 DH lines, which carried a chromosome with a recombination break point between the *HvBot1* gene copies and a chromosome with an intact Sahara 3771 gene cluster. Southern analysis identified homozygous recombinant progeny which likely retained only one of the copies of this gene. No sequence differences were identified between the *HvBot1* copy in the recombinant and those in the Sahara parent. We used this recombinant line to further investigate the impact of *HvBot1* duplication on expression. Levels of *HvBot1* expression in roots of homozygous recombinant F_3_ progeny were similar to expression levels in Clipper, while progeny with the full set of duplicated *HvBot1* copies showed similar levels of *HvBot1* expression to Sahara, and the heterozygous progeny had intermediate expression (Fig. 2).

We also assessed the function of each of the variants by heterologous expression in yeast (*Saccharomyces cereviseae*). With the exception of the Haruna Nijo allele (*Bot1.d*), all variants conferred a similar level of tolerance to media containing high concentrations of B when expressed in yeast (Panel A in Additional file 3: Figure S2). Clones containing the Haruna Nijo variant were no more tolerant to high B than empty vector control yeast, indicating that one or both of the residue substitutions in the Haruna Nijo HvBot1 allele disrupted protein function. We mutated each of the residues separately in the Sahara HvBot1 ORF and tested the two mutations in a second yeast expression experiment, which demonstrated that Leucine at position 234 in HvBot1 is critical for function (Fig. 1c). By contrast, the Thr541Met substitution had no effect on transporter function. The cultivars Haruna Nijo and Amagi Nijo which carry the Haruna Nijo HvBot1 allele are intolerant to high B when grown in hydroponics. Root B concentrations were similar to Clipper, and suggested that no net efflux of B from the roots was occurring in either cultivar (Panel B in Additional file 3: Figure S2).

We developed a KASP™ marker to track a SNP in exon 11 unique to Sahara (*wri57*; Additional file 4: Table S2, with marker location shown in Fig. 1a). The previously reported *xBot1* marker [12] (location shown in Fig. 1a) was designed around a SNP that is not specific to the Sahara *HvBot1* allele, but is also common to the Alexis, Morex, WI4304 and Haruna Nijo alleles (Fig. 1a).

### Genetic variation at the 6H locus (*HvNIP2;1*)

**Fig. 3:**
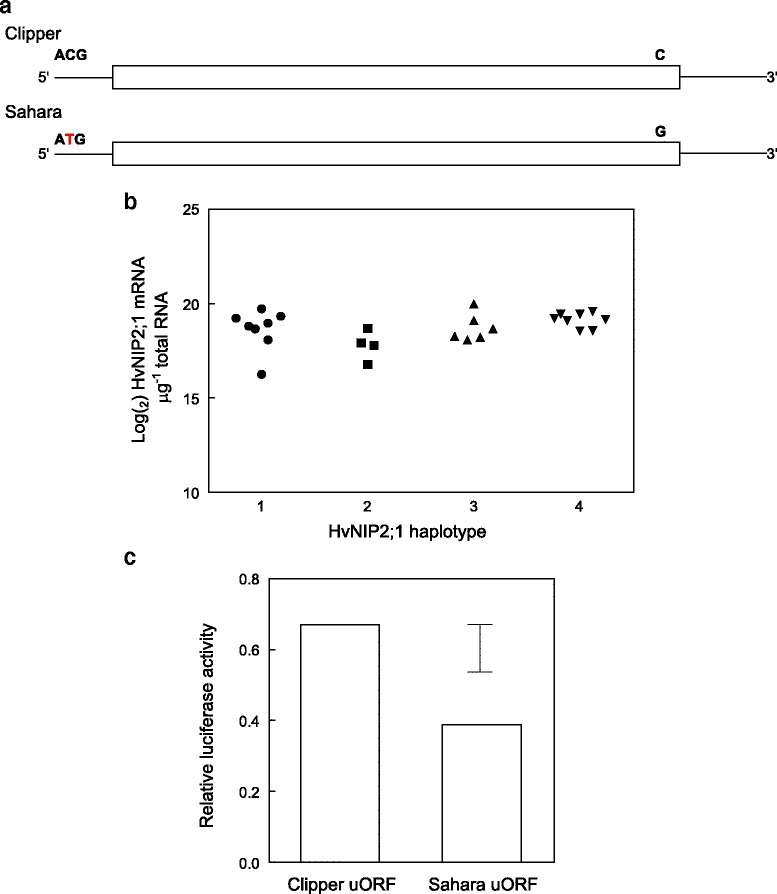
Genetic variation in barley at the 6H B tolerance locus (*HvNIP2;1*). **a** Schematic of *HvNIP2;1* coding sequences from Clipper and Sahara showing positions of two identified SNPs. No other sequence variation was found among 68 barley genotypes. A KASP^TM^ marker assay was designed against the Sahara-specific 5’UTR SNP. **b** Expression levels of *HvNIP2;1* determined by q-RT-PCR, in roots of seedlings of barley genotypes classified into four haplotypes on the basis of Southern and cDNA sequence analyses: Class 1, Clipper RFLP, ORF and uORF; 2, California Mariout RFLP, Clipper ORF and uORF; 3, Sahara RFLP and ORF/ Clipper uORF; 4, Sahara RFLP, ORF and uORF. Refer to Additional file 8: Table S5 for a list of barley genotypes included in each class. Transcript levels were normalised using the RNA housekeeping genes for tubulin, GAPDH and HSP70. **c** Relative translation efficiencies of a luciferase gene driven by an SP6 RNA polymerase promoter, when 5’UTR sequences from Clipper and Sahara *HvNIP2;1* are inserted immediately upstream of luciferase. Translation efficiency was measured as luciferase activity by luminescence, and is expressed relative to the activity of SP6 control luciferase DNA. Six transcription/translation reactions were set up for each construct, and four luciferase assays recorded for each reaction. Bar represents the 95 % confidence interval for the difference between Clipper and Sahara 5’UTR sequences

Initial Southern analysis of barley germplasm using a probe designed against *HvNIP2;1* identified three patterns of hybridisation. Sequencing of the coding regions of *HvNIP2;1* indicated that the *HvNIP2;1* ORF is very highly conserved; a single synonymous SNP occurs between the alleles from Clipper and Sahara 3771 (Fig. 3a). The Clipper sequence variant was found in just over half of all germplasm included for sequencing. Expression levels for *HvNIP2;1* in barley roots did not differ either between the two sequence variants or between the three allelic groups identified by genomic Southern analysis (Fig. 3b).

A second SNP, unique to the Sahara accessions and located 44 bp upstream of the ATG start codon in the 5’ UTR, was identified in *HvNIP2;1*. At this position the thymidine base in Sahara creates a short, upstream ORF (uORF) encoding a translatable peptide of thirteen residues immediately upstream of, and in frame with the *HvNIP2;1* ORF (Fig. 3a). We hypothesised that this uORF in Sahara may interfere with HvNIP2;1 translation, and be the principal mechanism for reduced function of HvNIP2;1 leading to reduced B permeability and a greater tolerance to high B. The 133 bp 5’UTR sequences from Clipper and Sahara *HvNIP2;1* were cloned into the SP6 vector, immediately upstream of the firefly (*Photinus pyralis*) luciferase gene driven by the SP6 RNA polymerase promoter. *In vitro* transcription/translation reactions with each construct demonstrated that the Sahara 5’ UTR sequence inhibited translation of the luciferase reporter gene by 43 % relative to the Clipper 5’ UTR sequence (Fig. 3c).

Interestingly, the uORF SNP was found only in Sahara 3771 and the other eight Sahara accessions, suggesting that tolerance to high soil B attributable to this locus is very rare. We developed a KASP™ marker to track the Sahara uORF SNP in *HvNIP2;1* (*wri59*; Additional file 4: Table S2).

### Genetic variation at the 3H and 2H loci

The genetic determinants at these loci are unknown. However, a member of the *Bot* B transporter gene family, *HvBot2*, is located within the region of the 3H QTL controlling relative root length at high B and is considered to be a likely candidate gene [15] ([GenBank:KR817581] lists the coding sequence for *HvBot2* from Clipper). The *HvBot2* sequence in Sahara 3771 is disrupted by a large deletion (approx. 5.1 kB) beginning at the 3’ end of the CDS, which may result in a loss of function. We designed two sets of primers, to amplify within and across the deletion, respectively (Additional file 5: Table S3). Discriminating PCR determined that with the exception of the nine Sahara accessions, the *HvBot2* CDS was intact in all tested barley genotypes. A KASP™ marker was developed to track the Sahara 3771 deletion (*wri58*; Additional file 4: Table S2).

The 2H QTL for B tolerance controls leaf symptom expression in the Clipper X Sahara 3771 population and is located close to the centromere [11]. Fine mapping in this region is difficult due to low recombination frequency. Furthermore, within the Clipper X Sahara 3771 DH population the QTL was poorly defined, largely because leaf symptoms could not be accurately assessed in lines carrying Sahara alleles at the other B tolerance loci. Additional background genetic effects might also contribute to an unclear phenotype in the DH lines. We identified seven DH lines that were recombinant within the 2H interval and backcrossed them to Clipper. BC_1_F_2_ plants retaining the recombinant Sahara haplotype across the 2H region but with Clipper alleles at the 3H, 4H and 6H loci were selected by genotyping. Evaluation of leaf symptoms of B toxicity in BC_1_F_3_ progeny clearly defined the interval for the 2H QTL to approximately 10.3 cM, between the markers GBMS0160 and Bmag381 (Additional file 6: Fig. S3, Panel A). We tested the markers contig888 and G9-138C in an attempt to characterise the barley genotypes at the 2H QTL region. G9-138C appeared to associate with a B tolerance phenotype. For this marker, the Sahara accessions and the line Ethiopia 756 shared a similar restriction pattern (Additional File 6: Fig. S3, Panel B).

### Prevalence and impact of B tolerance alleles in Australian barley varieties

A set of 80 Australian barley varieties included in National Variety Trial testing between 2008 and 2012 were screened for allele type at each of the four known B tolerance loci, leaf symptoms of B toxicity, and leaf B concentrations when grown in a glasshouse with an elevated supply of B. The data for each variety included in the screen are provided in Additional file 7: Table S4. A small number of varieties were heterogeneous for either the *HvNIP2;1* or *HvBot1* alleles, and were separated into sub-lines. The most common *HvBot1* allele amongst the Australian barley varieties was the Clipper allele (67 genotypes). The Morex (10 genotypes) and Alexis (7 genotypes) alleles were also relatively common. None of the genotypes contained the Sahara *HvNIP2;1* allele on chromosome 6H or the Sahara variant of *HvBot2* on chromosome 3H.

**Fig. 4:**
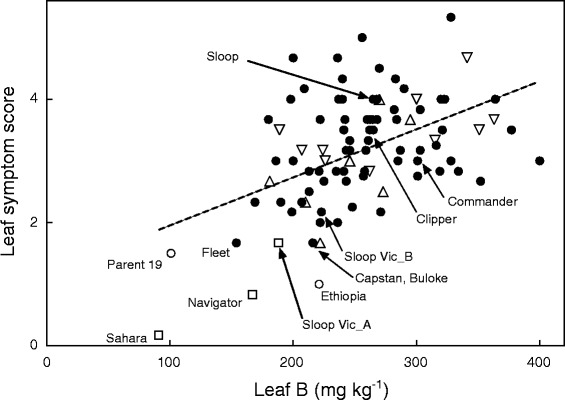
Relationship between the severity of B toxicity-induced leaf necrosis and leaf B concentration for a set of current Australian barley varieties. Symptoms were assessed visually three times during the growth of plants to full maturity and an average score determined (*0 = no necrosis; 6 = severe necrosis*). The penultimate leaves from five tillers were sampled at mid-grain fill for each measurement of leaf B concentration. *HvBot1* allele type for each variety is indicated (closed circles = Clipper; open circles = Sahara (*single-copy*); open squares = Sahara (*multi-copy*); open upward triangles = Alexis; open downwards triangles = Morex). The dashed line indicates a weak positive, linear correlation between the two parameters (R^2^ = 0.235)

Though few in number, genotypes in the study carrying single or multiple copies of the Sahara *HvBot1* allele all had both low leaf B concentrations and few symptoms of B toxicity-induced leaf necrosis (Fig. 4; Additional file 7: Table S4). The remaining genotypes showed no clear relationship between allele type at *HvBot1* and either leaf symptom score or leaf B concentrations. Similarly, we found no clear relationship of either B toxicity parameter to breeding origin (Additional file 7: Table S4). Overall there was only a weak positive relationship between leaf symptoms of B toxicity and leaf B concentration (R^2^ = 0.235; Fig. 4), indicating that mechanism (s) other than exclusion of shoot B may be contributing to B tolerance in Australian barleys.

**Fig. 5:**
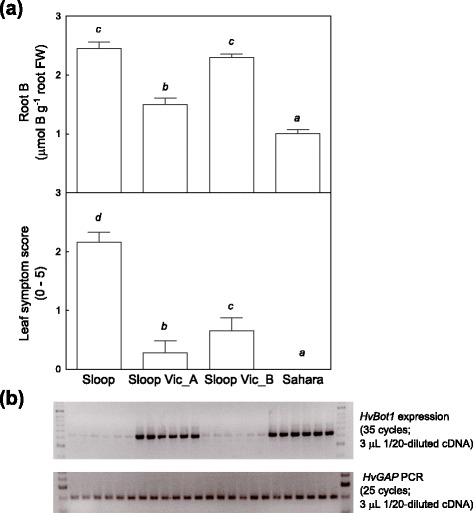
Contribution of Sahara-derived B tolerance alleles on chromosomes 2H and 4H to B tolerance traits in the barley cultivar Sloop Vic. **a** Root B concentrations (*upper panel*) and leaf symptoms of B toxicity (*lower panel*) in the parental genotypes Sloop and Sahara, and two Sloop Vic sublines. Subline Sloop Vic_A contains the multi-copy Sahara *HvBot1* allele on chromosome 4H, while Sloop Vic_B contains the Sloop (*Clipper*) *HvBot1* allele. Both sublines carry the 2H B tolerance allele from Sahara. Seedlings were grown for 13 days in solution culture (11 days at 3 mM B). Within each panel, italicised letters above the bars denote significant differences between genotypes (Tukey’s multiple comparisons test; *P* = 0.05) **b** Semi-quantitative RT-PCR analysis of *HvBot1* gene expression in roots of Sloop, Sloop Vic_A, Sloop Vic_B and Sahara seedlings grown for 11 days at 3 mM B. Duplicate reactions were run for each of three cDNA samples for each genotype, and appear alongside each other in the image. The lower panel shows *HvGAP* fragment amplification, to indicate template concentration variability

Two Australian cultivars, Navigator and Sloop Vic, have Sahara 3771 in their pedigrees and were found to contain the Sahara-derived *HvBot1* gene duplication. Both cultivars have high levels of *HvBot1* gene expression, and show tolerance to high soil B, with Sloop Vic bred specifically for this purpose. Sloop Vic was found to be genetically heterogeneous; within a single packet of seed we also identified seedlings carrying the Clipper *HvBot1* allele. We designated sublines Sloop Vic_A and Sloop Vic_B, as carrying the Sahara *HvBot1* duplication or the Clipper *HvBot1* allele, respectively. Interestingly, while Sloop Vic_A had elevated levels of *HvBot1* expression and reduced root B concentrations relative to Sloop Vic_B and the parent cultivar Sloop, both Sloop Vic sublines showed low leaf symptoms of B toxicity (Figs. 4 and 5). Further genotyping revealed that both Sloop Vic_A and Sloop Vic_B have retained a segment from Sahara chromosome 2H, carrying the gene or genes controlling leaf symptom expression.

**Fig. 6:**
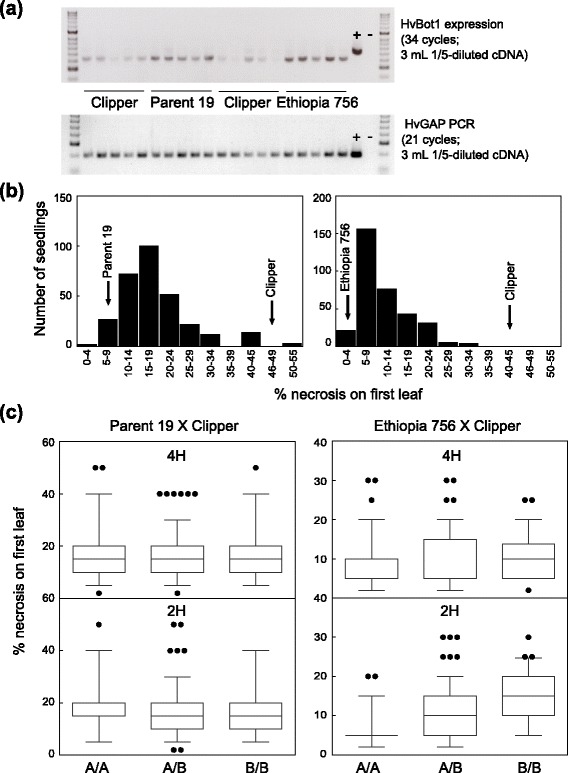
A single copy of the Sahara *HvBot1* allele on chromosome 4H does not contribute to B tolerance in barley. **a** Semi-quantitative RT-PCR analysis of *HvBot1* expression in roots of Clipper, Parent 19 and Ethiopia 756 seedlings. Each sample is derived from a single seedling grown in nutrient solution containing an additional 3 mM B for 15 days. The lower panel shows *HvGAP* fragment amplification, to indicate template concentration variability. + = positive control using *HvGAP* PCR product as template; − = water control. **b** Distribution of percentage leaf necrosis in F_2_ progeny derived from the crosses Parent 19 × Clipper (*left panel; N = 294*) and Ethiopia 756 × Clipper (*right panel; N = 337*). Seedlings were grown for 15 days in nutrient solution containing 3 mM B. Average necrosis for each parent is also indicated. **c** Leaf necrosis of F_2_ plants in the two populations segregating for *HvBot1* allele (*chromosome 4H; upper panels*) and a KASP^TM^ marker linked to the 2H B tolerance locus (lower panels). In all panels, the Parent 19 or Ethiopia allele is represented by A, and the Clipper allele represented by B. The boundaries of the boxes indicate 75^th^ and 25^th^ centiles, lines within mark the median, bars above and below the boxes indicate 90^th^ and 10^th^ centiles, and outliers are shown as circles. The percentage variation (R^2^) explained in each population by marker genotype is also shown

In the varietal screen we included genotypes Parent 19 and Ethiopia 756, both containing a single copy of the Sahara *HvBot1* allele. The two genotypes have been identified in Australian breeding programs to have good tolerance to high soil B. Parent 19 was provided to us by the University of Adelaide’s Barley Breeding Program, while Ethiopia 756 was suggested for testing by breeders in Western Australia. In our screening Parent 19 and Ethiopia 756 also had both low leaf B concentrations and few leaf symptoms of B toxicity (Fig. 4). Although q-RT-PCR and semi-q-RT-PCR experiments did not detect sizeable differences in *HvBot1* expression between the Clipper and single-copy Sahara variants (Figs. 1b and 6a), we could not rule out a link between the presence of a single copy of the Sahara *HvBot1* allele and a B tolerance phenotype. To determine the contribution of the Sahara *HvBot1* allele to B tolerance in these genotypes, we generated F_2_ populations from the crosses Parent 19 × Clipper and Ethiopia 756 × Clipper, and assessed leaf symptoms of B toxicity (Fig. 6b). Genetic segregation analysis did not reveal linkage between B tolerance determined by leaf symptom expression and the *HvBot1* allele in either population (Fig. 6c). We developed a discriminating KASP™ assay (*ABC02403*; Additional file 5: Table S3) around sequence polymorphism between Clipper and the B tolerant parents of these populations, within the region on chromosome 2H associated with B tolerance in Sahara. Using this marker assay, we found an association between genotype at 2H and B tolerance in the Ethiopia 756 × Clipper F_2_ segregants (Fig. 6c). F_2_ seedlings with a homozygous Ethiopia 756 genotype had, on average, 6.3 % leaf necrosis on the first leaf. This was less than half the level of necrosis estimated on leaves of seedlings with a homozygous Clipper genotype, and a reduction of 33 % compared to heterozygous seedlings (Fig. 6c). In the Parent 19 × Clipper F_2_ population there was no association between the 2H KASP™ marker assay and a B tolerance phenotype.

In our varietal screen, several other barley cultivars also showed both low leaf symptoms of B toxicity and low leaf B concentrations, including Fleet, Capstan and Buloke (Fig. 4; Additional file 7: Table S4). We phenotyped a barley DH population (233 lines) derived from reciprocal crosses of Fleet with Commander. QTL analysis failed to reveal an association between leaf symptoms of B toxicity and any of the known B tolerance loci (data not shown). Thus, the chromosomal regions responsible for B tolerance observed in Parent 19, Fleet and other cultivars with lower levels of tolerance remain unknown, and do not appear to correspond to any of the B tolerance QTL found in Sahara 3771.

## Discussion

The identities of two genes conferring tolerance to high soil B in barley have been determined [12, 13, 16]. One encodes HvNIP2;1, a transport protein belonging to the NIP family of aquaporins, and is found on chromosome 6H. The other is on chromosome 4H and encodes a different kind of transporter, HvBot1. The tolerant alleles for both genes are present in a set of barley accessions from North Africa named Sahara, including the well-studied genotype Sahara 3771. A genetic study using a population derived from an intolerant barley variety, Clipper, and Sahara 3771, identified four chromosome locations associated with B tolerance, with all of the favourable alleles coming from Sahara 3771 [11]. In this study, we investigated the prevalence of these four alleles in barley germplasm, and the allelic diversity of *HvBot1* and *HvNIP2;1*. Amongst the studied germplasm, we found that the Sahara lines contain a rare set of tolerance alleles, including a tandem duplication of *HvBot1* (4H), a critical 5’UTR SNP in the coding sequences of *HvNIP2;1* (6H), a deletion in the gene *HvBot2* that is located under the 3H tolerance locus, and a rare haplotype across the 2H QTL region.

Tolerance due to HvBot1 (chromosome 4H) was originally attributed to either greater functionality of the transporter as a result of sequence differences between the Sahara and Clipper *HvBot1* coding sequences, or higher levels of expression [12]. In addition to promoter sequence differences, tandem duplication of *HvBot1* in the genome of Sahara has created an estimated four copies. Our study indicates that this duplication occurred relatively recently in barley evolution, and is rare. Southern analysis showed that the duplication is only present in Sahara 3771, eight other genotypes also accessioned with the Sahara name, and two Australian cultivars that contain Sahara 3771 in their pedigree. Sequence analysis of many genotypes in this study also revealed that the original published *HvBot1* sequence for Clipper contained a sequencing error. There are in fact no residue differences between the alleles from Clipper and Sahara 3771, and both alleles confer a similar level of B tolerance to yeast when heterologously expressed (Fig. 1). All *HvBot1* sequences lodged with GenBank have been amended and updated accordingly. Thus, differences between the two alleles are entirely attributable to expression level differences.

We identified seven *HvBot1* CDS alleles in total. Only one of these, found in two Japanese cultivars Haruna Nijo and Amagi Nijo, showed reduced function when expressed in yeast. This is due to a residue substitution at position 234 in the non-functional Haruna Nijo allele, of histidine for leucine (Fig. 1c). All single-copy *HvBot1* alleles were expressed at similarly low levels, including the single-copy Sahara *HvBot1* allele (Fig. 1b). Genomic sequencing of a number of single-copy Sahara *HvBot1* genotypes failed to identify any sequence differences between the single-copy and multi-copy Sahara alleles, or between copies present in Sahara. Moreover, a line derived from the Clipper X Sahara 3771 DH population and with a recombination within the *HvBot1* gene cluster, showed a dependency of *HvBot1* gene expression level on the number of Sahara *HvBot1* copies present (Fig. 2). We conclude that the increased expression of *HvBot1* found in Sahara is due to the duplication of this gene. A number of other specific examples of gene copy number expansion occurring through evolution have been described in plant genomes, which have also resulted in increased gene expression [17–19]. Recent whole genome analyses have revealed that copy number variation is common in plants, and appears to be biased towards genes involved in abiotic and biotic stress responses [20, 21].

Further, we propose that duplication of the Sahara *HvBot1* allele is responsible for B tolerance at the 4H locus. Segregation analysis in two independent F_2_ barley populations (Parent 19 × Clipper; Ethiopia 756 × Clipper) failed to demonstrate linkage between the presence of a single copy of the Sahara *HvBot1* allele and B tolerance as determined by leaf symptom expression (Fig. 6). By contrast, in the original genetic study the duplicated *HvBot1* allele from Sahara was associated with leaf symptom development, as well as a number of other traits associated with B tolerance [11].

We also examined diversity in coding sequence and gene expression level for *HvNIP2;1*, the gene underlying a major QTL in Sahara 3771 on chromosome 6H [13], revealed that the *HvNIP2;1* sequences were highly conserved amongst the studied germplasm. We identified only two SNPs in coding sequence of this gene; one synonymous SNP that was present in around half of the genotypes examined and could not be related to a B tolerance phenotype, and a second SNP in the 5’UTR that was unique to the set of nine Sahara accessions (Fig. 3a). We propose that the 5’UTR SNP is causative of B tolerance in Sahara. An *in vitro* transcription/translation assay showed reduced luciferase activity in the presence of the 5’UTR derived from Sahara *HvNIP2;1* (Fig. 3c), suggesting that a small uORF created by the Sahara 5’UTR SNP interferes with translation of *HvNIP2;1*. In our previous study we detected a modest reduction in *HvNIP2;1* transcript levels in root tissues of B-treated Sahara 3771 compared to Clipper [13]. However, in broadening the analysis of expression to a wider range of genotypes, we were not able to reproduce these differences (Fig. 3b). Future experiments to quantify active protein levels in roots of Sahara and other, intolerant barley genotypes will help to confirm if the uORF in Sahara inhibits *HvNIP2;1* translation. Upstream ORFs are common elements of plant transcript sequences and have been shown to mediate translation for a number of genes (reviewed in [22]), including transcription factors [23, 24] and enzymes involved in polyamine biosynthesis [25].

It has been suggested that when using Sahara as a breeding parent, there may be linkage drag of unfavourable traits together with the 4H allele, or pleiotropic effects of the 4H tolerance allele in different backgrounds [26, 27]. However, we identified two released barley varieties carrying the 4H allele from Sahara, Navigator and Sloop Vic (Additional file 7: Table S4), demonstrating there are unlikely to be severe penalties in either yield or quality traits associated with the introgression of the *HvBot1* gene duplication. We investigated by genotype analysis the extent of the Sahara-derived 4H segment in Navigator (WI4262; released 2009), and estimate it to cover a region of at least 14 cM. While developed for cultivation in higher rainfall, long-season environments not typically associated with B toxicity, Navigator consistently shows low levels of leaf symptom expression when challenged with high B (this study and [28]).

The cultivar Sloop Vic was specifically selected for B tolerance [29]. We identified genetic heterogeneity for the 4H locus in this variety (Fig. 5), but also the presence of a chromosomal segment from Sahara that includes the 2H B tolerance allele. The two other tolerance alleles from Sahara (3H and 6H) were not retained in Sloop Vic. Our findings suggest that the 2H allele is highly useful for imparting B tolerance. We also identified another unadapted six-row barley, Ethiopia 756, as an alternative source of the 2H tolerance allele. Ethiopia 756 shares the same haplotype for a number of markers across the 2H interval with Sahara, including a rare *G9-138C* allele. Segregation analysis in a population derived from Ethiopia 756 crossed with Clipper revealed an association between the critical 2H region in Ethiopia 756 and leaf symptoms of B toxicity (Fig. 6). In an alternative population derived from Parent 19 and Clipper the same region was not linked with B tolerance.

## Conclusions

This study of allelic diversity for the four known boron tolerance loci revealed that Sahara 3771 has a unique set of boron tolerance genes, rare amongst germplasm. It allowed us to identify the causative features of two of the genes responsible for B tolerance in Sahara. These are a tandem duplication of *HvBot1* and a short, upstream open reading frame in the coding sequences of *HvNIP2;1*. Our findings facilitate the development of markers for tracking B tolerance in barley with greater specificity, and we designed three KASP™ marker assays for tracking tolerance derived from Sahara. Our detection of the 2H locus in Ethiopia 756 is the first reported validation of the Sahara 3771 B tolerance QTL in another population.

The genetic locations controlling B tolerance in Parent19, as well as lower levels of tolerance in cultivars such as Fleet, Buloke and Capstan that were observed in our study and in the field by others [6, 7], remain unknown; selection for B tolerance derived from these sources can only be made on the basis of phenotype. However, the genotypes Ethiopia 756 and Sloop Vic may be used in breeding programs as sources of B tolerance attributable to a significant QTL for leaf symptom expression on chromosome 2H, and may be valuable alternatives to Sahara.

## Methods

### Plant material

Seed for most genotypes of barley (*Hordeum vulgare* L.) was obtained from either the Australian Grains Genebank or from our own collections. Seed of Parent 19 and a number of ICARDA genotypes showing B tolerance in the field was provided by Drs Jason Eglinton and Stewart Coventry, The University of Adelaide’s Barley Breeding Program. The varieties Compass, Flinders, Grange, Henley, LaTrobe, SY Rattler and Westminster were obtained from Dr Hugh Wallwork (South Australian Research and Development Institute), and Macumba was obtained from Amanda Box (The University of Adelaide).

### Allele diversity analyses

Seeds were germinated on moist filter paper in the laboratory. After 4–7 days, whole roots and shoots from five uniform seedlings for each genotype were pooled and snap-frozen in liquid nitrogen, for RNA and DNA extractions, respectively. Pooling from a number of individual seedlings facilitated the detection of genetic heterogeneity within lines. The 65 genotypes included for allele diversity analysis are listed in Additional file 1: Table S1.

RNA was extracted using TRIzol (Invitrogen). For cDNA synthesis, we used Superscript III Reverse Transcriptase (Invitrogen); samples for quantitative real-time reverse transcription polymerase chain reaction (q-RT-PCR) were DNase-treated prior to cDNA synthesis using DNA-Free (Ambion, USA). Coding sequence for *HvBot1* and *HvNIP2;1* was amplified from cDNA obtained from 68 genotypes, and Sanger sequenced at the Australian Genome Research Facility (AGRF; Adelaide). q-RT-PCR analysis of expression of *HvBot1* and *HvNIP2;1* was performed as previously described [30], using gene-specific primers (Additional file 5: Table S3). For some experiments, semi-quantitative reverse transcription PCR (semi-q-RT-PCR) was performed on cDNAs, where we used *HvGAP* amplification (Additional file 5: Table S3) to indicate between-sample variation in template concentration.

Genomic DNA was isolated using phenol-chloroform extraction, digested with the restriction enzyme *Dra* I and the products separated by gel electrophoresis before transfer to a nylon membrane for Southern hybridisation using standard methods. Membranes were probed with radio-labelled nucleic acid fragments of the B tolerance genes *HvBot1* and *HvNIP2;1,* and the putative tolerance gene *HvBot2* (co-locating with a tolerance QTL on chromosome 3H). Several probes located within the region of the 2H B tolerance QTL were also utilised. Probe details are included in Additional file 5: Table S3. Membranes were stripped with a 0.1 % sodium dodecyl sulphate (SDS), 2 mM ethylenediaminetetraacetic acid (EDTA) solution heated to boiling point between each sequential hybridisation.

### Heterologous expression and *in vitro* transcription/translation experiments

Open reading frames for each of the identified *HvBot1* alleles were PCR-amplified from cDNA prepared from roots of the barley genotypes Sahara 3771, Clipper, Haruna Nijo, WI4304, Alexis and Tadmor using primers listed in Additional file 5: Table S3, and products were cloned in the Gateway entry vector *pCR8* (Invitrogen, Carlsbad, CA, USA). Sequences of the inserts were verified by Sanger sequencing before recombining into the destination vector pYES3.DEST (Invitrogen) for yeast expression. The Sahara *HvBot1* expression vector was also used as a template for *in vitro* site-directed mutagenesis (Quikchange II Site-Directed Mutagenesis kit, Stratagene, La Jolla CA, USA) using specific primer pairs detailed in Additional file 5: Table S3. Two variants of HvBot1 were created, each with a single residue substitution of either Leu234His or Thr541Met. Both substitutions are present in the non-functional Haruna Nijo HvBot1 allele. Experiments for functional assessment of the HvBot1 alleles and variants in yeast (*Saccharomyces cerevisiae*) were performed as previously described [31].

5’UTR sequences from *HvNIP2;1* genes of Clipper and Sahara were amplified from cDNA using primers listed in Additional file 5: Table S3. The PCR products and SP6 control luciferase vector DNA (Promega, USA) were digested with restriction enzymes *Not* I and *Bam* HI. Digested products were then cloned into the linearised SP6 vector, between the SP6 RNA polymerase promoter and the firefly (*Photinus pyralis*) luciferase gene. Insert sequences were confirmed by Sanger sequencing. We then used a TnT Coupled Wheat Germ Extract System (Promega, USA) to perform *in vitro* transcription/translation reactions using SP6 control DNA and the modified vector. We made six reactions for each construct. Translation of luciferase protein for each reaction was measured as luciferase activity, by recording luminescence using a POLARstar Optima plate reader (BMG LabTech) programmed to perform multiple reads on each sample at 1 s intervals for a total of approximately 2 min. All plotted data were linear with slopes close to zero, and were extrapolated to time 0 to determine initial rates of activity. Four luminescence assays were recorded for each transcription/translation reaction.

### Screening of current varieties

Seeds were sown into 50:50 UC mix: coco peat potting medium, in 25 cm diameter pots without replication. Following germination, each pot was thinned to three uniform seedlings. Plants were grown through to maturity during spring, in a temperature-controlled greenhouse (day/night average temperatures of 23 °C/19 °C) with natural lighting. Pots were moved during the growing period to avoid positional effects on light interception or transpiration, which might influence the development of symptoms of B toxicity. Plants were watered regularly with tap water, and fertilised twice with a multi-element, slow-release fertiliser. Tap water delivered to the University of Adelaide’s Waite Campus between August and December 2013 contained B concentrations of 0.1–0.5 mg B L^−1^, which was sufficient to induce B toxicity-attributable leaf necrosis in intolerant barley varieties in this experiment. We have consistently observed B toxicity symptoms in barley plants grown across the Waite Campus since 2012, co-incident with elevated B concentrations in tap water (data not supplied). In this experiment, symptoms of B toxicity were assessed visually three times during the growth of plants to full maturity and an average score determined (0 = no necrosis; 6 = severe necrosis). The penultimate leaves from five tillers were sampled at mid-grain fill, oven-dried and chopped finely with scissors. A sub-sample of dried leaf tissue was acid-digested for analysis of B concentration by Radial View Inductively Coupled Plasma-Optical Emission Spectrometry (ICP-OES) [32]. Selected varieties were grown hydroponically with high B using a nutrient solution described in [12]. B concentrations in root tissues of these plants were determined using an azomethine-H colorimetric assay [33].

### Development of KASP™ markers to detect the Sahara 3771 allele at the *HvBot1*, *HvBot2* and *HvNIP2;1* loci

KASP™ markers were designed based on polymorphisms between Sahara 3771 and B intolerant cultivars at the B tolerance loci *HvBot1*, *HvBot2* and *HvNIP2;1* (Additional file 4: Table S2). The markers *wri57* and *wri59* (for *HvBot1* and *HvNIP2;1*, respectively) assay SNP polymorphisms between the Sahara accessions and B intolerant lines, while the *wri58* marker (for *HvBot2*) assays a deletion present only in Sahara accessions. All primer sets were designed using Kraken (LGC Limited, London, UK) with default parameters. Assays were validated in the parental lines and a set of varieties for which alleles were previously determined using an automated SNPLine system (LGC Limited, London, UK).

### F_2_ segregant analysis of B toxicity symptoms

Crosses were made using two barley lines with low levels of leaf B toxicity symptoms as female parents (Parent 19 and Ethiopia 756), and the intolerant variety Clipper as the male parent. F_1_ hybrids from these crosses were confirmed by genotyping using the *xBot1* CAPS marker (Additional file 5: Table S3). F_2_ progeny were germinated on moist filter paper, and transferred to aerated nutrient solution containing 3 mM B. Seedlings were grown for 15 d in a growth chamber (18 °C/15 °C and 50 %/70 % humidity day/night settings, with a 12 h photoperiod and light intensity of approx. 200 mol m^−2^ s^−1^ at plant height). Individual seedlings were then scored for percentage necrosis on the first leaf, and sampled for DNA isolation using a freeze-dried method of extraction [34]. Material was genotyped at the 4H and 2H B tolerance loci using the KASP™ assays *wri57* (Additional file 4: Table S2) and *ABC02403* (Additional file 5: Table S3), respectively. The 2H KASP™ marker was designed and validated as discriminating between Clipper and the B tolerant parents, and verified by genotyping of selected recombinant lines to reside within the defined QTL interval. Association between genotype and phenotype for each population and each of the 4H and 2H regions was analysed by separate one-way ANOVA.

## Availability of supporting data

The data sets supporting the results of this article are included within the article and its additional files. Nucleic acid sequences described in this research have been lodged with GenBank: [GenBank:EF660435, GenBank:EF660437, GenBank:KR605456, GenBank:KR605457, GenBank:KR605458, GenBank:KR605459, GenBank:KR605460, GenBank:KR605461, GenBank:KR817581].

## Additional files

Additional file 1: Table S1. Barley genotypes used for initial characterisation of allele diversity at each of four boron tolerance loci (PDF 233 kb) Additional file 2: Figure S1. Nine barleys accessioned with the name Sahara contain the rare *HvBot1* gene duplication. (a) Southern analysis of allele type and gene duplication at *HvBot1*, for a representative set of barley genotypes. Lane 1, Clipper; 2, Sahara 3771; 3; Alexis; 4, Amagi Nijo; 5, Arapiles; 6, Atlas; 7, Barque; 8, California Mariout; 9, Chebec; 10, CM72; 11, Commander; 12, Flagship; 13, Fleet; 14, Franklin; 15, Gairdner; 16, Galleon; 17, Golden Promise; 18, Halcyon; 19, Haruna Nijo; 20, Clipper; 21, Sahara 3771; 22, Keel; 23, Morex; 24, Mundah; 25, Sahara 3763; 26, Sahara 3764; 27, Sahara 3765; 28, Sahara 3766; 29, Sahara 3767; 30, Sahara 3768; 31, Sahara 3769; 32, Sahara 3770, 33, Schooner; 34, Skiff; 35, Sloop; 36, Steptoe; 37, Tadmor; 38, Tokak. Genomic DNA was digested with *Dra* I, and probed with a [^32^P]-labelled nucleic acid fragment of *HvBot1* derived from Clipper (Additional File 5: Table S3). (b) Leaf symptom scores (upper panel) and shoot B concentrations (lower panel) of nine Sahara barley accessions grown in nutrient solutions with 5 mM additional B (PDF 1165 kb) Additional file 3: Figure S2. The Haruna Nijo variant of HvBot1 is non-functional. (a) Growth of yeast expressing *HvBot1* alleles relative to either an empty vector control (top two plates only) or clones expressing the Sahara *HvBot1* allele, on low B media (left) or at high B (right). Three independent clones for each allele were cultured on each plate; clones are positioned vertically and spots across the plates represent 10 L of 10^−0^, 10^−1^, 10^−2^ and 10^−3^ dilutions from a starting culture containing approximately 3 × 10^7^ cells L^−1^. Photographs were taken after 2 d (low B) or 4 d (20 mM B) incubation at 30 °C. With the exception of the Haruna Nijo allele, all variants conferred a similar level of tolerance to high B in yeast to the Sahara *HvBot1* allele. (b) Concentration of B in roots of barley seedlings (cvs. Clipper, Haruna Nijo, Amagi Nijo and Sahara) grown for 11 days in hydroponics supplemented with 3 mM B (N = 5, ± sd). The genotypes Haruna Nijo and Amagi Nijo carry the non-functional Haruna Nijo *HvBot1* allele at the 4H B tolerance QTL (PDF 369 kb) Additional file 4: Table S2. KASP™ marker assays designed around Sahara-specific gene coding sequence features for *HvBot1*, *HvNIP2;1* and *HvBot2*. These markers are suitable for tracking the introgression of the 4H, 6H and 3H B tolerance alleles, respectively, from the Sahara accessions (PDF 386 kb) Additional file 5: Table S3. Primers, PCR markers and probes used in this study (PDF 350 kb) Additional file 6: Figure S3. Resolution of the 2H interval containing a QTL for leaf symptom expression in a Clipper X Sahara 3771 barley doubled haploid (DH) population. (a) The graph shows leaf symptom scores (0 = no symptoms; 5 = severe leaf necrosis) for 2 – 4 BC_1_F_2_ families of each of seven lines derived from selected DH lines backcrossed to Clipper and selected for intolerant alleles at each of the 3H, 4H and 6H B tolerance QTL. Each of the lines was also selected for homozygosity of the 2H recombinant haplotype. Symptom scores for the parental genotypes (Clipper and Sahara 3771), and eight additional DH lines with unequivocal 2H genotypes and Clipper alleles at the other loci are shown for comparison (open symbols). The schematic below shows genotypes for each of the lines across the 2H interval, with open and shaded bars representing Clipper- and Sahara-derived chromosome segments, respectively. Markers within the interval are in bold type, and span a region of 10.3 ± 2.9 cM. The KASP™ marker designed to discriminate between Clipper and Parent 19 or Ethiopia 756 and used for genotyping F_2_ segregants from Parent 19 × Clipper and Ethiopia 756 × Clipper populations in Fig. 6 is shown in red type. (b) Southern analysis of *G9-138C* allele type for a representative set of barley genotypes, including the Sahara accessions (lanes 6–13) and Ethiopia 756 (lane 23). Lanes 1 and 20; Clipper. Lanes 2 and 21; Sahara 3771 (PDF 640 kb) Additional file 7: Table S4. Breeding origin, genotype at *HvBot1*, and B tolerance phenotype of 80 current or recent Australian barley varieties grown to maturity in a glasshouse with an elevated supply of B. Breeding lines Parent 19 and Ethiopia 756 were also included in the screen, as well as Sahara 3771 and Clipper control genotypes. All varieties had a Clipper (B-intolerant) allele at chromosomes 6H and 3H, while at 2H only Sloop Vic_A, Sloop Vic_B and Ethiopia 756 possessed the tolerant (Sahara) allele. Varieties are listed in order of increasing severity of leaf symptoms expression. Symptoms were assessed visually three times during the growth of plants to full maturity and an average score determined (0 = no necrosis; 6 = severe necrosis). The penultimate leaves from five tillers were sampled at mid-grain fill for determination of leaf B concentration (PDF 464 kb) Additional file 8: Table S5. Barley genotypes included in each (a) *HvBot1* allele class and (b) *HvNIP2;1* haplotype class, for gene expression analyses data presented in Figs. 1b and 3b, respectively (PDF 301 kb)
