# Supplementary material for: Diversity in boron toxicity tolerance of Australian barley (Hordeum vulgare L.) genotypes
Source: BMC Plant Biol. 2015 Sep 26;15:231. doi: 10.1186/s12870-015-0607-1 (PMC4584011; doi:10.1186/s12870-015-0607-1)
Supplement: Additional file 1: Table S1. — Barley genotypes used for initial characterisation of allele diversity at each of four boron tolerance loci (PDF 233 kb) [file 12870_2015_607_MOESM1_ESM.pdf]

Table S1. Barley genotypes used for initial characterisation of allele diversity at each of four boron tolerance loci.

Genotype	Source/Origin
Abyssinian 21	Ethiopia
Alexis	Germany
Amagi Nijo	Japan
Anadolu	Turkey
Arapiles	Australia (VIC)
Atlas	US?
Barque	Australia (SA)
BulBul	Turkey
BulBul 89	Turkey
Buloke	Australia (VIC)
California Mariout	US (California)
Chebec	Australia (SA)
CI-3576	North Africa
Clippper	Australia (SA)
CM67	US (California)
CM72	US (California)
Commander	Australia (SA)
Ethiopia 756	Ethiopia
Flagship	Australia (SA)
Fleet	Australia (SA)
Franklin	Australia (TAS)
Gairdner	Australia (WA)
Galleon	Australia (SA)
Golden Promise	England
Halcyon	England
Hamidye	Turkey
Harrington	Canada
Haruna Nijo	Japan
ICARDA009	Barley breeders (University of Adelaide)
ICARDA061	Barley breeders (University of Adelaide)
ICARDA075	Barley breeders (University of Adelaide)
ICARDA079	Barley breeders (University of Adelaide)
ICARDA080	Barley breeders (University of Adelaide)
ICARDA083	Barley breeders (University of Adelaide)
ICARDA084	Barley breeders (University of Adelaide)
ICARDA085	Barley breeders (University of Adelaide)
ICARDA087	Barley breeders (University of Adelaide)
ICARDA088	Barley breeders (University of Adelaide)
ICARDA098	Barley breeders (University of Adelaide)
Keel	Australia (SA)
Morex	US (Minnesota)
Mundah	Australia (WA)
Navigator	Australia (SA)

Parent 18	Barley breeders (University of Adelaide)
Parent 19	Barley breeders (University of Adelaide)
Sahara 3763	North Africa or Algeria
Sahara 3764	North Africa or Algeria
Sahara 3765	North Africa or Algeria
Sahara 3766	North Africa or Algeria
Sahara 3767	North Africa or Algeria
Sahara 3768	North Africa or Algeria
Sahara 3769	North Africa or Algeria
Sahara 3770	North Africa or Algeria
Sahara 3771	North Africa or Algeria
Schooner	Australia (SA)
Shannon	Australia (TAS)
Skiff	Australia (SA)
Sloop	Australia (SA)
Steptoe	US (Washington)
Tadmor	Syria
Tarm 92	Turkey
Tokak	Turkey
Vlamingh	Australia (WA)
WI4304	Australia (SA)
WI4330	Australia (SA)
