# Supplementary figures and images for: Diversity in boron toxicity tolerance of Australian barley (Hordeum vulgare L.) genotypes
Source: BMC Plant Biol. 2015 Sep 26;15:231. doi: 10.1186/s12870-015-0607-1 (PMC4584011; doi:10.1186/s12870-015-0607-1)

(a)

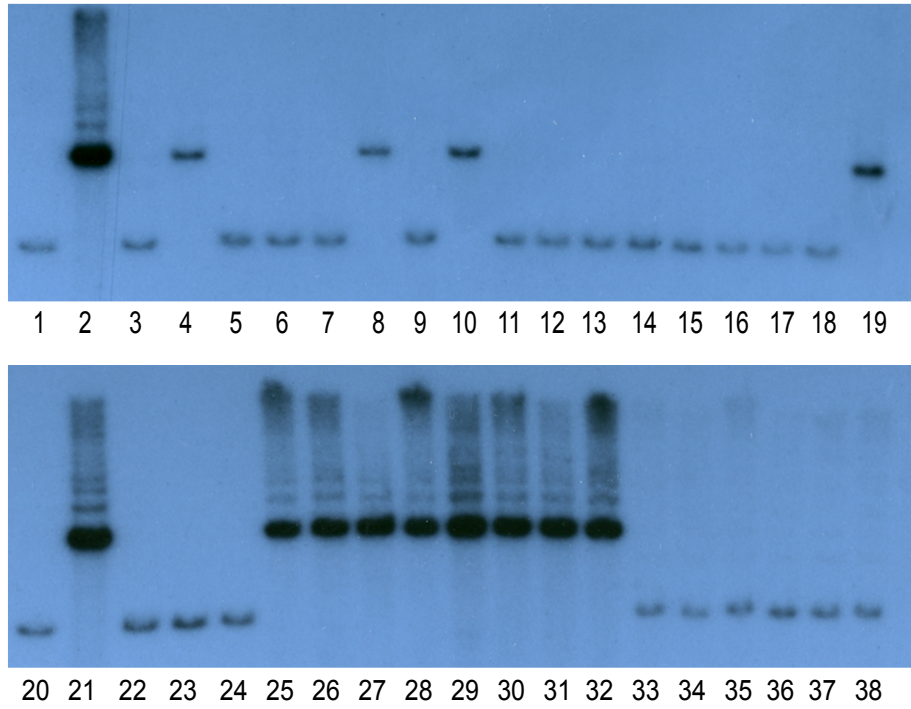

(b)

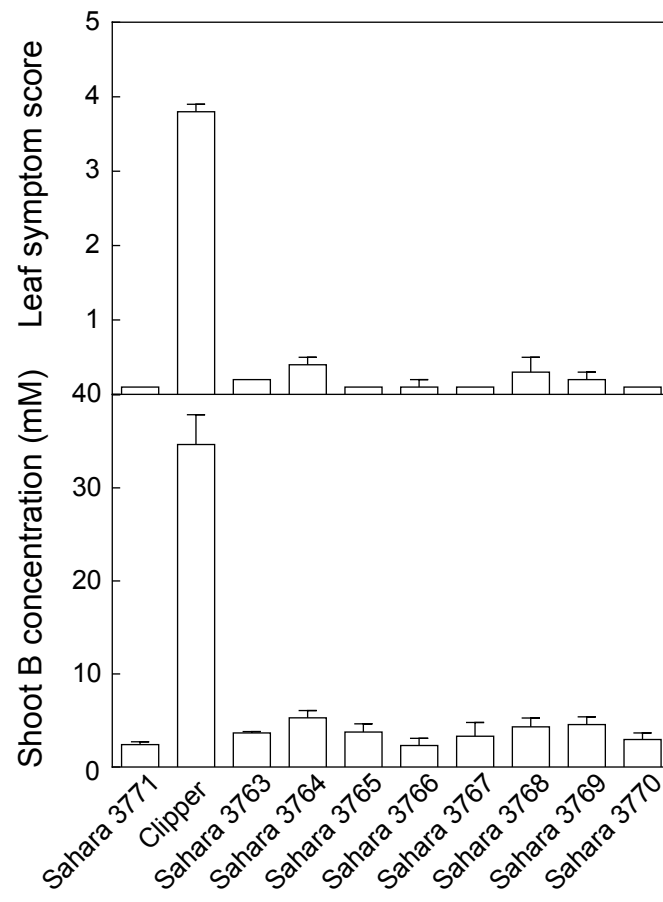

Supplement: Additional file 2: Figure S1. — Nine barleys accessioned with the name Sahara contain the rare HvBot1 gene duplication. (a) Southern analysis of allele type and gene duplication at HvBot1, for a representative set of barley genotypes. Lane 1, Clipper; 2, Sahara 3771; 3; Alexis; 4, Amagi Nijo; 5, Arapiles; 6, Atlas; 7, Barque; 8, California Mariout; 9, Chebec; 10, CM72; 11, Commander; 12, Flagship; 13, Fleet; 14, Franklin; 15, Gairdner; 16, Galleon; 17, Golden Promise; 18, Halcyon; 19, Haruna Nijo; 20, Clipper; 21, Sahara 3771; 22, Keel; 23, Morex; 24, Mundah; 25, Sahara 3763; 26, Sahara 3764; 27, Sahara 3765; 28, Sahara 3766; 29, Sahara 3767; 30, Sahara 3768; 31, Sahara 3769; 32, Sahara 3770, 33, Schooner; 34, Skiff; 35, Sloop; 36, Steptoe; 37, Tadmor; 38, Tokak. Genomic DNA was digested with Dra I, and probed with a [32P]-labelled nucleic acid fragment of HvBot1 derived from Clipper (Additional File 5: Table S3). (b) Leaf symptom scores (upper panel) and shoot B concentrations (lower panel) of nine Sahara barley accessions grown in nutrient solutions with 5 mM additional B (PDF 1165 kb) [file 12870_2015_607_MOESM2_ESM.pdf]

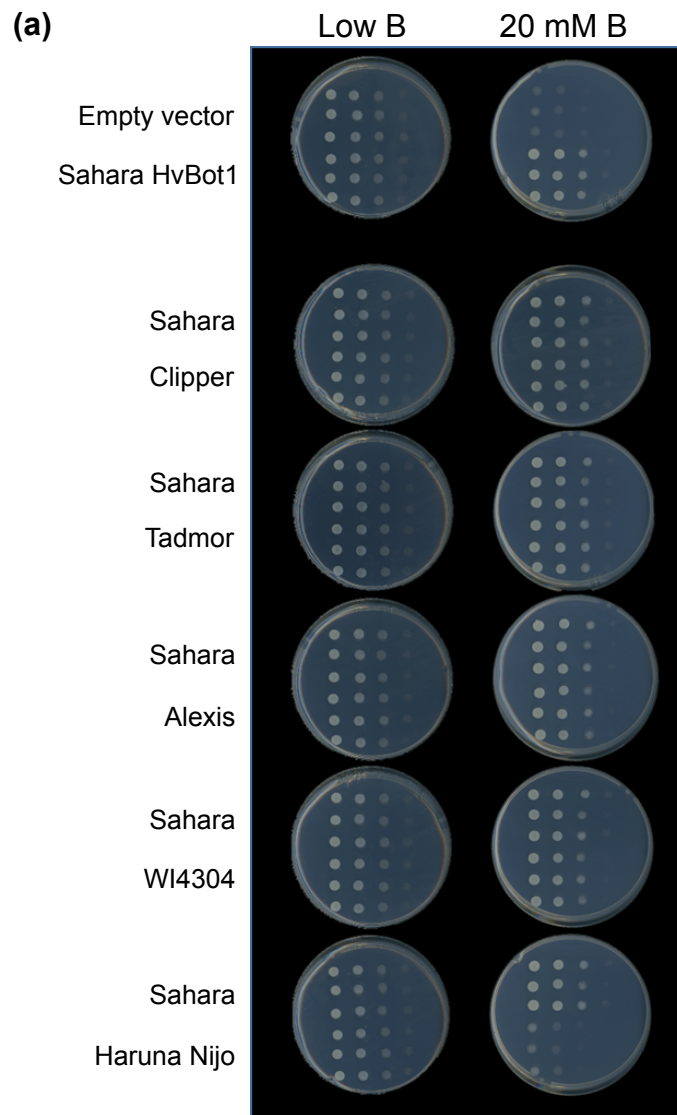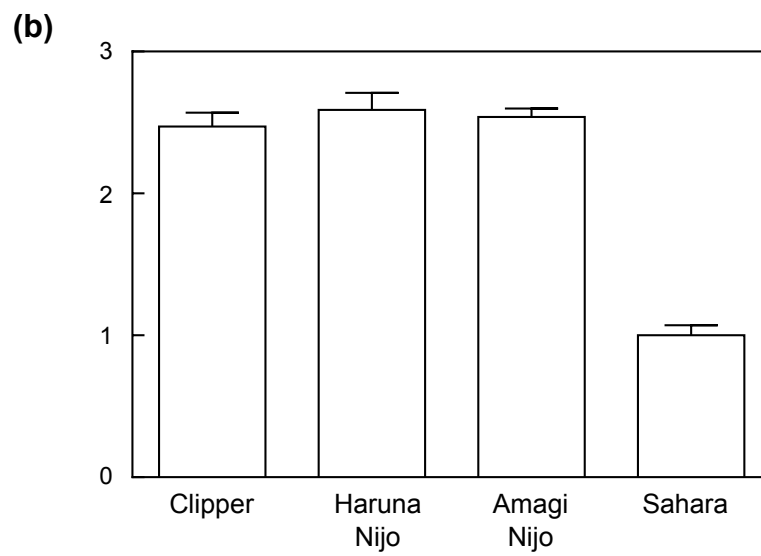

Supplement: Additional file 3: Figure S2. — The Haruna Nijo variant of HvBot1 is non-functional. (a) Growth of yeast expressing HvBot1 alleles relative to either an empty vector control (top two plates only) or clones expressing the Sahara HvBot1 allele, on low B media (left) or at high B (right). Three independent clones for each allele were cultured on each plate; clones are positioned vertically and spots across the plates represent 10 L of 10−0, 10−1, 10−2 and 10−3 dilutions from a starting culture containing approximately 3 × 10^7^ cells L−1. Photographs were taken after 2 d (low B) or 4 d (20 mM B) incubation at 30 °C. With the exception of the Haruna Nijo allele, all variants conferred a similar level of tolerance to high B in yeast to the Sahara HvBot1 allele. (b) Concentration of B in roots of barley seedlings (cvs. Clipper, Haruna Nijo, Amagi Nijo and Sahara) grown for 11 days in hydroponics supplemented with 3 mM B (N = 5, ± sd). The genotypes Haruna Nijo and Amagi Nijo carry the non-functional Haruna Nijo HvBot1 allele at the 4H B tolerance QTL (PDF 369 kb) [file 12870_2015_607_MOESM3_ESM.pdf]

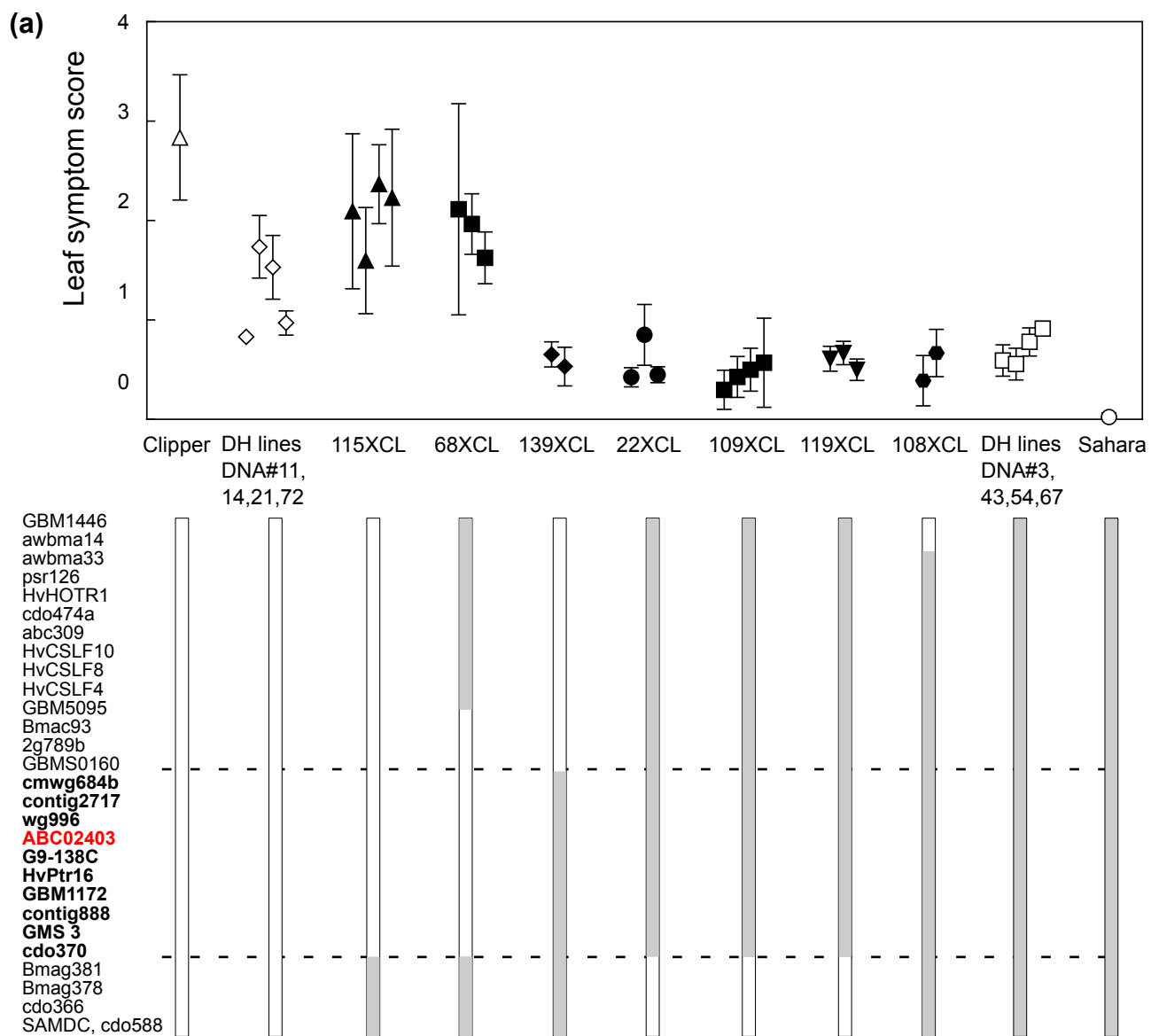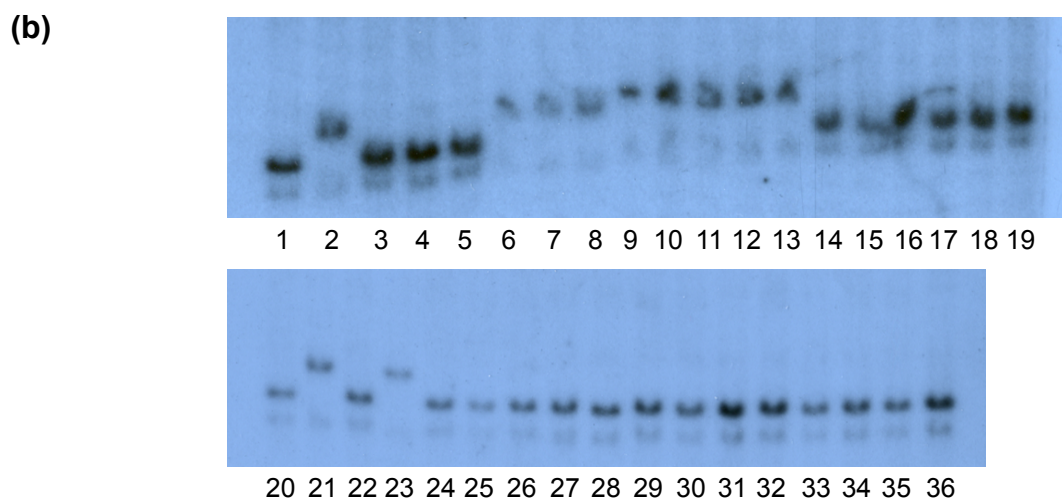

Supplement: Additional file 6: Figure S3. — Resolution of the 2H interval containing a QTL for leaf symptom expression in a Clipper X Sahara 3771 barley doubled haploid (DH) population. (a) The graph shows leaf symptom scores (0 = no symptoms; 5 = severe leaf necrosis) for 2 – 4 BC1F2 families of each of seven lines derived from selected DH lines backcrossed to Clipper and selected for intolerant alleles at each of the 3H, 4H and 6H B tolerance QTL. Each of the lines was also selected for homozygosity of the 2H recombinant haplotype. Symptom scores for the parental genotypes (Clipper and Sahara 3771), and eight additional DH lines with unequivocal 2H genotypes and Clipper alleles at the other loci are shown for comparison (open symbols). The schematic below shows genotypes for each of the lines across the 2H interval, with open and shaded bars representing Clipper- and Sahara-derived chromosome segments, respectively. Markers within the interval are in bold type, and span a region of 10.3 ± 2.9 cM. The KASP™ marker designed to discriminate between Clipper and Parent 19 or Ethiopia 756 and used for genotyping F2 segregants from Parent 19 × Clipper and Ethiopia 756 × Clipper populations in Fig. 6 is shown in red type. (b) Southern analysis of G9-138C allele type for a representative set of barley genotypes, including the Sahara accessions (lanes 6–13) and Ethiopia 756 (lane 23). Lanes 1 and 20; Clipper. Lanes 2 and 21; Sahara 3771 (PDF 640 kb) [file 12870_2015_607_MOESM6_ESM.pdf]
