# Supplementary material for: Diversity in boron toxicity tolerance of Australian barley (Hordeum vulgare L.) genotypes
Source: BMC Plant Biol. 2015 Sep 26;15:231. doi: 10.1186/s12870-015-0607-1 (PMC4584011; doi:10.1186/s12870-015-0607-1)
Supplement: Additional file 4: Table S2. — KASP™ marker assays designed around Sahara-specific gene coding sequence features for HvBot1, HvNIP2;1 and HvBot2. These markers are suitable for tracking the introgression of the 4H, 6H and 3H B tolerance alleles, respectively, from the Sahara accessions (PDF 386 kb) [file 12870_2015_607_MOESM4_ESM.pdf]

Table S2. KASP™ marker assays designed around Sahara-specific gene coding sequence features for *HvBot1*, *HvNIP2;1* and *HvBot2*. These markers are suitable for tracking the introgression of the 4H, 6H and 3H B tolerance alleles, respectively, from the Sahara accessions.

Marker	Gene	Primer sequences (5'-3')
wri57	*HvBot1* (4H)	wri57_A1:GAAGGTGACCAAGTTCATGCTCACGCAGCACAGCATCCTTCAAA<br>wri57_A2:GAAGGTCGGAGTCAACGGATTACGCAGCACAGCATCCTTCAAG<br>wri57_C1:CAGGCTGATTCTGTTGACAAGGACTT
wri58	*HvBot2* (3H)	wri58_A1:GAAGGTGACCAAGTTCATGCTGTCGTGAGTTGATCCTATTCCACA<br>wri58_A2:GAAGGTCGGAGTCAACGGATTCGTGAGTTGATCCTATTCCACC<br>wri58_C1:CTTGCAATGATCCTAGCTATGCATATCAA<br>wri58_C2:CAAAACCCCTCTCTTTCCGAGAAGAA
wri59	*HvNIP2;1* (6H)	wri59_A1:GAAGGTGACCAAGTTCATGCTAGCCGCCGAGAGCTCG<br>wri59_A2:GAAGGTCGGAGTCAACGGATTGCTAGCCGCCGAGAGCTCA<br>wri59_C1:TTCCCCCTGCCACTACCGAGTA
