# Supplementary material for: Diversity in boron toxicity tolerance of Australian barley (Hordeum vulgare L.) genotypes
Source: BMC Plant Biol. 2015 Sep 26;15:231. doi: 10.1186/s12870-015-0607-1 (PMC4584011; doi:10.1186/s12870-015-0607-1)
Supplement: Additional file 5: Table S3. — Primers, PCR markers and probes used in this study (PDF 350 kb) [file 12870_2015_607_MOESM5_ESM.pdf]

Table S3. Primers, PCR markers and probes used in this study.

Marker name	Primer sequences (5'-3')	Reference /Notes
**CAPS markers**		
xBot1 (4H)	F: GATCCTTTTCCCGCTACCTTTCTTC<br>R: GTACCGTGCATATTATCACGGAAAG<br>restriction endonuclease: *Pvu* I	[1]<br>GenBank Accession No. BV723959
xBM178 (4H)	F: TCGTCATCCCCTTCACCTGCCTCTG<br>R: AGCTGGAAGGTGTTTGAACTGCAGC<br>restriction endonuclease: *Hin*f I	[1]<br>GenBank Accession No. BV723962
xBM6001 (*HvNIP2;1* uORF SNP) (6H)	F: CTGCTAGTAGTATCTGCCACCTCGC<br>R: CCAGGGGAAATGGCGGAAGATG<br>Restriction endonuclease: *Rsa* I	This study<br>(Also used for sequencing)
xHvNIP2;1 (3' SNP) (6H)	F: CTCTGGCTCTACTTCCTGGG<br>R: GAGTTTACAAGTGACACACACGCACACG<br>Restriction endonuclease: *Btg* I	[2]
**RFLP markers**		
HvBot1 fragment (4H)	F: GATAGAACAATGGCCCAGGACCGAC<br>R: TCAAACAGAACAAAGCCAGGCACAC	[1]
HvNIP2;1 fragment (6H)	F: GGACGGATCTCCCAGTGTGTGTCCT<br>R: GTCGTTTGCACACATGAACAGAGCG	[2]
G9-138C (2H)	CTCTCCCTGGCGCGCTTCTAGGAGGAAGGAAGGGAGAT<br>GGGATTGAGGGAAGAGAGGAGTGAGGATCAGGCAGGG<br>ATCAAGGGAGAGGCTAGGGTTTCGATAGGTGGGGGCGG<br>ATGGGCCTTTGGCCCACTTTGCTATAGTGTGGGATCCCTC<br>TCTCTCACCACTTTCCATTAGCAGGAAAAAAAGAAAGAA<br>AGAAAAGAGAGGAAAAAAAGTTAGAGAAGGAATTTAGG<br>CATGGTGTTCATTTCACTCGACTA	(Complete probe sequence given)
contig888 (2H)	F: GGAGGTGATCGCCATCAACG<br>R: TTGAGGTTGGGGAGCACCAG	This study
**PCR markers**		
HvBot2_CL (3H)	F: AGGCTAAGCAACCTGCTGCAATCCTTAC<br>R: CAGAATCGTCATCAAAGCCTCGGACATC	This study; 1280 bp (no product in Sahara)
HvBot2_SA (3H)	F: GTATGAATTCAGCTTGGGTTCTTGG<br>R: TGAGTTCATCATATGATTCTTCCG	This study; 622 bp (product only in Sahara; spans large deletion)
GMS003 (2H)	F: TTTCAGCATCACACGAAAGC<br>R: TTGCATGCATGCATACCC	[3]<br>approx. 144 bp
**KASP™ markers**		
ABC02403 (2H)	A1: GAAGGTGACCAAGTTCATGCTAGGCTTGACTCATTAAGCCCGC<br>A2: GAAGGTCGGAGTCAACGGATTAAGGCTTGACTCATTAAGCCCGT<br>C1: CGAGCATGGTTTTCTGCTTGTTAATCTTA	This study

**q-RT-PCR and semi-q-RT-PCR on cDNAs**		
HvBot1 fragment	F: CAACATGAACACACATTGGAGGAAG<br>R: AAGAACGACTGCCTGAGGATTTCCC	[1]<br>267 bp
HvNIP2;1 fragment	F: CCGTCCTTGGCACGCTCAG<br>R: GCAGGATCAGACGGGGATG	This study;<br>167 bp
HvGAP	F: GTGAGGCTGGTGCTGATTACG<br>R: TGGTGCAGCTAGCATTTGAGAC	197 bp
**PCR for sequencing**		
HvBot1 CDS	F: CACACCACACCACAACAGCTGCTTC<br>R: GTACCGTGCATATTATCACGGAAAG	This study;<br>2241 bp
HvNIP2;1 CDS	F: CTGCCACTACCGAGTATGAGCTCTC<br>R: TACCAAGGACACACACTGGGAGATC	This study; uORF SNP underlined; 1011 bp
HvNIP2;1 gDNA	F: CTGCTAGTAGTATCTGCCACCTCGC<br>R: CCAGGGGAAATGGCGGAAGATG	This study; 684 bp (spans uORF SNP)
**Cloning experiments**		
HvBot1 allele cloning for yeast expression	F: AAAATGTCTGATCTACTGAGGAACCCCTTCAAGGGA<br>R: TCACACGCTTGGCTGAACG	Yeast consensus sequence [4] underlined; 2007 bp
In vitro site-directed mutagenesis of Sahara *HvBot1*	F: CTGTATGGTATAGGATGGCATAGGAGCTTCATTGCCG<br>R: CGGCAATGAAGCTCCTATGCCATCCTATACCATACAG<br><br>F: GTCGGTGTCTTCGAGAATGATATATGTCTTTACGATC<br>R: GATCGTAAAGACATATATCATTCTCGAAGACACCGAC	Leu234His. Underlined base indicates altered sequence<br>Thr541Met. Underlined base indicates altered sequence
HvNIP2;1 5'UTR cloning for uORF analysis	F: TATAGCGGCCGCAAGCGAGCTAAGCCAGCCAG<br>R: ATTGGATCCTGTTCACCACGAACTACGTAC	*Not* I and *Bam* HI restriction sites underlined; 133 bp

References

[1] Sutton T, Baumann U, Hayes J Collins NC, Shi B-J, Schnurbusch T et al. Boron toxicity tolerance in barley arising from efflux transporter amplification. Science 2007;318:1446-1449.

[2] Schnurbusch T, Hayes JE, Hrmova M, Baumann U, Ramesh SA, Tyerman SD et al. Boron toxicity tolerance in barley through reduced expression of the multifunctional aquaporin HvNIP2;1. Plant Physiol. 2010;153:1706-1715.

[3] Graingenes website: http://wheat.pw.usda.gov

[4] Hamilton R, Watanabe CK, de Boer HA. Compilation and comparison of the sequence context around the AUG start codons in *Saccharomyces cerevisiae* mRNAs. Nucleic Acids Res. 1987;15:3581-3593.
