# Supplementary material for: Diversity in boron toxicity tolerance of Australian barley (Hordeum vulgare L.) genotypes
Source: BMC Plant Biol. 2015 Sep 26;15:231. doi: 10.1186/s12870-015-0607-1 (PMC4584011; doi:10.1186/s12870-015-0607-1)
Supplement: Additional file 7: Table S4. — Breeding origin, genotype at HvBot1, and B tolerance phenotype of 80 current or recent Australian barley varieties grown to maturity in a glasshouse with an elevated supply of B. Breeding lines Parent 19 and Ethiopia 756 were also included in the screen, as well as Sahara 3771 and Clipper control genotypes. All varieties had a Clipper (B-intolerant) allele at chromosomes 6H and 3H, while at 2H only Sloop Vic_A, Sloop Vic_B and Ethiopia 756 possessed the tolerant (Sahara) allele. Varieties are listed in order of increasing severity of leaf symptoms expression. Symptoms were assessed visually three times during the growth of plants to full maturity and an average score determined (0 = no necrosis; 6 = severe necrosis). The penultimate leaves from five tillers were sampled at mid-grain fill for determination of leaf B concentration (PDF 464 kb) [file 12870_2015_607_MOESM7_ESM.pdf]

Table S4. Breeding origin, genotype at *HvBot1*, and B tolerance phenotype of 80 current or recent Australian barley varieties grown to maturity in a glasshouse with an elevated supply of B. Breeding lines Parent19 and Ethiopia 756 were also included in the screen, as well as Sahara 3771 and Clipper control genotypes. All varieties had a Clipper (B-intolerant) allele at chromosomes 6H and 3H, while at 2H only Sloop Vic_A, Sloop Vic_B and Ethiopia 756 possessed the tolerant (Sahara) allele. Varieties are listed in order of increasing severity of leaf symptoms expression. Symptoms were assessed visually three times during the growth of plants to full maturity and an average score determined (0 = no necrosis; 6 = severe necrosis). The penultimate leaves from five tillers were sampled at mid-grain fill for determination of leaf B concentration.

Variety	Breeding origin	*HvBot1* allele (chromosome 4H)	Symptoms score (0 – 6)	Leaf blade B (mg B kg^{-1} DW)
Sahara 3771	North Africa	Sahara (multi-copy)	0.2	91
Navigator	SA	Sahara (multi-copy)	0.8	167
Ethiopia 756	North Africa	Sahara (single copy)	1.0	221
Parent 19	ICARDA	Sahara (single copy)	1.5	101
Fleet	SA	Clipper	1.7	154
Sloop Vic_A[1.]	VIC	Sahara (multi-copy)	1.7	188
Capstan	SA	Clipper	1.7	216
Buloke	VIC	Alexis	1.7	222
Shepherd	QLD	Clipper	2.0	222
Westminster	UK	Clipper	2.0	236
Mackay	QLD	Clipper	2.2	199
Sloop Vic_B[1.]	VIC	Clipper	2.2	223
Henley	Europe/UK	Clipper	2.2	266
Brindabella_B[2.]	ACT/NSW	Clipper	2.3	248
Grange	Europe/UK	Clipper	2.3	169
Arapiles	VIC	Clipper	2.3	190
Barque	SA	Clipper	2.3	207
Scope	VIC	Alexis	2.3	210
Harrington	Canada	Clipper	2.5	213
Fathom_D[3.]	SA	Alexis	2.5	273
Franklin	TAS	Alexis	2.7	181
Wyalong	NSW	Clipper	2.7	225
Fairview	NZ	Clipper	2.7	243
Fathom_B[3.]	SA	Clipper	2.7	352
Finniss	SA	Clipper	2.8	301
Yagan	Mexico	Clipper	2.8	213
Compass	SA	Clipper	2.8	221
Galleon	SA	Clipper	2.8	235
Dampier	WA	Clipper	2.8	241
Fathom_A[3.]	SA	Clipper	2.8	258
Lockyer_B[4.]	WA	Morex	2.8	262
Keel	SA	Clipper	2.8	319
Bass	WA	Clipper	2.8	334

VT Admiral	SA	Clipper	3.0	186
Flagship	SA	Clipper	3.0	200
Yarra	VIC	Morex	3.0	226
Fathom_C[3.]	SA	Alexis	3.0	246
Fitzroy	VIC	Clipper	3.0	285
Commander	SA	Clipper	3.0	301
Binalong	NSW	Clipper	3.0	328
Onslow	WA	Morex	3.2	207
Yerong	NSW	Morex	3.2	224
Brindabella_A[2.]	ACT/NSW	Clipper	3.2	243
Flinders	WA	Clipper	3.2	246
Skipper	SA	Clipper	3.2	259
Molloy_A[5.]	WA	Clipper	3.2	287
Hindmarsh	VIC	Clipper	3.2	303
Macumba_B[6.]	SA	Clipper	3.3	389
Cowabbie	NSW	Clipper	3.3	246
Baudin	WA	Clipper	3.3	261
Merlin	US	Morex	3.3	315
Tilga	NSW	Morex	3.5	189
SY Rattler	Europe/UK	Clipper	3.5	241
Clipper	SA	Clipper	3.5	262
Schooner	SA	Clipper	3.5	265
Unicorn	Japan	Clipper	3.5	321
Macumba_A[6.]	SA	Morex	3.5	351
Tulla	NSW	Clipper	3.7	180
Chebec	SA	Clipper	3.7	222
Sloop SA	SA	Clipper	3.7	242
O'Connor	WA	Clipper	3.7	260
La Trobe	WA	Clipper	3.7	262
Lockyer_A[4.]	WA	Clipper	3.7	264
Grout	QLD	Clipper	3.7	268
Tallon	QLD	Clipper	3.7	284
Wimmera	SA	Alexis	3.7	295
Macquarie	TAS	Morex	3.7	363
Kaputar	NSW/QLD	Clipper	3.8	282
Grimmett	QLD	Clipper	3.8	303
Dash	VIC	Clipper	4.0	198
Yambla	NSW	Clipper	4.0	237
Oxford	UK	Clipper	4.0	240
Doolup	WA	Clipper	4.0	265
Sloop	SA	Clipper	4.0	268
Fitzgerald	WA	Alexis	4.0	270
Hannan	WA	Morex	4.0	300
Mundah	WA	Clipper	4.0	320
Skiff	SA	Clipper	4.0	323
Molloy_B[5.]	WA	Clipper	4.0	364

Dhow	SA	Clipper	4.2	209
Maritime	SA/WA	Clipper	4.2	290
Stirling	WA	Clipper	4.3	240
Tantangara	NSW	Clipper	4.3	283
Roe	WA	Clipper	4.5	270
Vlamingh	WA	Clipper	4.7	200
Torrens	SA	Clipper	4.7	236
Gairdner	WA	Morex	4.7	341
Urambie	NSW	Clipper	5.0	256
Hamelin	WA	Clipper	5.3	328

[1] Sloop Vic was sourced from the Australian Grains Genebank and separated into two sub-lines on the basis of genotype at 4H. Sloop Vic_A seedlings have the multi-copy Sahara *HvBot1* allele; Sloop Vic_B have a Clipper *HvBot1* allele.
[2] Our seed stock of Brindabella was separated into two sub-lines on the basis of (Brindabella_A earlier maturing), and allele type using the CAPS marker *xHvNIP2;1*.
[3] Our seed stock of Fathom was very mixed on the basis of genotyping at the 4H and 6H B tolerance loci, and was separated into four sub-lines.
[4] Lockyer was sourced from the Australian Grains Genebank and separated into two sub-lines on the basis of genotyping at the 4H locus.
[5] Our seed stock of Molloy was separated into two sub-lines based on differences in plant height and maturity (Molloy_A earlier maturing/taller stature; Molloy_B later maturing/shorter stature)
[6] Macumba was separated into two sub-lines on the basis of genotype at 4H and plant height (Macumba_B very short stature)
