# Supplementary material for: Diversity in boron toxicity tolerance of Australian barley (Hordeum vulgare L.) genotypes
Source: BMC Plant Biol. 2015 Sep 26;15:231. doi: 10.1186/s12870-015-0607-1 (PMC4584011; doi:10.1186/s12870-015-0607-1)
Supplement: Additional file 8: Table S5. — Barley genotypes included in each (a) HvBot1 allele class and (b) HvNIP2;1 haplotype class, for gene expression analyses data presented in Figs. 1b and 3b, respectively (PDF 301 kb) [file 12870_2015_607_MOESM8_ESM.pdf]

Table S5. Barley genotypes included in each (a) *HvBot1* allele class and (b) *HvNIP2;1* haplotype class, for gene expression analyses data presented in Figures 1b and 3b, respectively.

(a) *HvBot1* allele class	Genotypes
Single-copy Sahara	California Mariout, CM67, CM72, Parent 19
Multi-copy Sahara	Sahara accessions only
Clipper	Barque, Chebec, Clipper, Halcyon, Schooner, Tokak, WI4330, Shannon, ICARDA009, ICARDA075, ICARDA080, ICARDA087
Tadmor	Tadmor, ICARDA085, ICARDA088
Alexis	Alexis, Buloke, Franklin
Morex	Gairdner, Morex
WI4304	WI4304
Haruna Nijo	Haruna Nijo, Amagi Nijo
**(b) *HvNIP2;1* haplotype class**	
1.) Clipper RFLP, ORF and uORF	Amagi Nijo, Chebec, Clipper, Flagship, Golden Promise, Halcyon, Schooner, ICARDA075
2.) CM RFLP, Clipper ORF and uORF	California Mariout, Mundah, Sloop, Vlamingh
3.) Sahara RFLP and ORF, Clipper uORF	Gairdner, Parent 19, ICARDA080, ICARDA083, ICARDA085, ICARDA088
4.) Sahara RFLP, ORF and uORF	Sahara accessions only
